# Commercial Fucoidans from *Fucus vesiculosus* Can Be Grouped into Antiadipogenic and Adipogenic Agents

**DOI:** 10.3390/md16060193

**Published:** 2018-06-04

**Authors:** Ruth Medeiros Oliveira, Rafael Barros Gomes Câmara, Jessyka Fernanda Santiago Monte, Rony Lucas Silva Viana, Karoline Rachel Teodosio Melo, Moacir Fernandes Queiroz, Luciana Guimarães Alves Filgueira, Lila Missae Oyama, Hugo Alexandre Oliveira Rocha

**Affiliations:** 1 Departamento de Bioquímica, Universidade Federal do Rio Grande do Norte, Natal, Rio Grande do Norte 59.078-970, Brazil; ruth.oliveira@ifrn.edu.br (R.M.O.); jessykamonte@gmail.com (J.F.S.M.); rony_lucas@hotmail.com (R.L.S.V.); melo.krt@gmail.com (K.R.T.M.); moacirfqn@gmail.com (M.F.Q.); lucianagalves@hotmail.com (L.G.A.F.); 2 Instituto Federal de Educação, Ciência e Tecnologia do Rio Grande do Norte, Caicó, Rio Grande do Norte 59.300-000, Brazil; rafael_bgc@yahoo.com.br; 3 Escola Multicampi de Ciências Médicas, Universidade Federal do Rio Grande do Norte, Caicó, Rio Grande do Norte 59.300-000, Brazil; 4 Departamento de Fisiologia, Universidade Federal de São Paulo—Escola Paulista de Medicina, São Paulo 04023-060, Brazil; lmoyama@gmail.com

**Keywords:** 3T3-L1 cells, fucan, lipolytic, obesity, brown seaweed

## Abstract

*Fucus vesiculosus* is a brown seaweed used in the treatment of obesity. This seaweed synthesizes various bioactive molecules, one of them being a sulfated polysaccharide known as fucoidan (FF). This polymer can easily be found commercially, and has antiadipogenic and lipolytic activity. Using differential precipitation with acetone, we obtained four fucoidan-rich fractions (F0.5/F0.9/F1.1/F2.0) from FF. These fractions contain different proportions of fucose:glucuronic acid:galactose:xylose:sulfate, and also showed different electrophoretic mobility and antioxidant activity. Using 3T3-L1 adipocytes, we found that all samples had lipolytic action, especially F2.0, which tripled the amount of glycerol in the cellular medium. Moreover, we observed that FF, F1.0, and F2.0 have antiadipogenic activity, as they inhibited the oil red staining by cells at 40%, 40%, and 50%, respectively. In addition, they decreased the expression of key proteins of adipogenic differentiation (C/EBPα, C/EBPβ, and PPARγ). However, F0.5 and F0.9 stimulated the oil red staining at 80% and increased the expression of these proteins. Therefore, these fucoidan fractions have an adipogenic effect. Overall, the data show that F2.0 has great potential to be used as an agent against obesity as it displays better antioxidant, lipolytic and antiadipogenic activities than the other fucoidan fractions that we tested.

## 1. Introduction

*Fucus vesiculosus* is a brown seaweed commonly found in coastal wetlands, in temperate or cold waters of the Atlantic and Pacific oceans. It was shown that *F. vesiculosus* intake helps women with abnormal menstrual cycles, and health problems associated with their periods [1]. Other author also reported that consumption of this seaweed promotes a decrease in body weight [2,3]. This seaweed has various active elements in its composition, of which fucoidan is one of the best known.

The presence of fucoidan in *F. vesiculosus* was demonstrated in 1913, and was initially called fucoidin [4]. Years later, it was suggested that the term be changed to fucoidan [5].

The structure of fucoidan (FF) from *F. vesiculosus* was last reviewed by Patankar et al. [6]. It was suggested that it possesses a central core formed by α-L-fucose (1,3)-linked, sulfated at C4. In addition, several branching points (every two or three fucose residues) were present in α-(1,2) or α-(1,4)-linked, on the main chain.

Currently, it is easy to acquire fucoidan from *F. vesiculosus*, as a multinational company sells it commercially. In part, this may explain the large amount of research and the number of activities ascribed to this sulfated polysaccharide, including antilipemic [7,8], and antiadipogenic activities [9,10].

Adipogenesis is a process of cell differentiation in which mesenchymal stem cells differentiate into adipocytes, with this process initially involving a stage where cells “compromise” with the adipocyte line [11]. In the next step, differentiation occurs, and the pre-adipocytes develop into mature adipocytes. The process of differentiation is complex and involves the participation of hundreds of proteins, although two proteins particularly play a crucial role in this event: C/EBPα, a protein of the CCAAT-enhancer binding protein class, and PPARγ, a peroxisome proliferator activated receptor [12].

Although pre-adipocyte primary cultures are an important tool for understanding the mechanisms of adipocyte differentiation, these cells have low mitogenic capacity and lose their ability to differentiate over time under culture conditions [13]. Therefore, the development of studies on adipogenesis is carried out mainly by the use of cellular models, such as the murine 3T3-L1 and 3T3-F442A (pre-adipocyte) lines. These cells, when stimulated to differentiate into adipocytes, follow the same metabolic pathways of differentiation of mesenchymal cells [14].

By using a differentiation cocktail based on insulin, dexamethasone, isobutylmethylxanthine, and fetal bovine serum, it is possible to obtain mature adipocytes from a 3T3-L1 culture. The actions of these compounds result in the initial events of differentiation, represented by the expression of CCAAT-enhancer binding proteins [13,14,15]. Afterwards, the cells return to their cell cycles, undergo clonal expansion in a regulated manner, and enter a terminal differentiation process by activation of PPARγ and C/EBPα [16]. Besides, the differentiation of these cells takes place in precisely controlled sequential stages: cell cycle arrest, clonal expansion, and differentiation (first phase and second phase of activation of transcriptional factors), by activating hundreds of previously silenced genes [15].

Studies with pre-adipocyte 3T3-L1 cells showed that fucoidan from *F. vesiculosus* inhibits adipogenesis. Real-time polymerase chain reaction (PCR) data showed that fucoidan reduced mRNA expression of C/EBPα and PPARγ by 22.6% and 17.6%, respectively [9].

FF from *F. vesiculosus* can be fractionated, with certain fractions showing very similar activities to each other [10]. Moreover, Nishino et al. [17] reported that some fucoidan fractions showed much greater activity than others did. However, antiadipogenic activity across different fucoidan populations has not yet been evaluated. With this in mind, we obtained four different fucoidan-rich fractions of commercial fucoidan from *F. vesiculosus* and, assessed them for their adipogenic activity.

## 2. Results

### 2.1. Obtaining Different Fractions of Fucoidan (FF) 

Using differential precipitation with acetone, we obtained four fractions from FF. These were called F0.5, F0.9, F1.1, and F2.0 corresponding to 4.5%, 35.2%, 22.0% and 38.3% of the material, respectively (Table 1). Chemical analysis and sulfated polysaccharide yield are summarized in Table 1. Data show that mannose and glucose were not found in the samples, whereas fucose, glucuronic acid, galactose and xylose were found in all samples. The data also showed fucose was the major component present in all fractions, whereas the relative amounts of other monosaccharides vary according to the fraction. Thus, the relative amounts of these sugars vary according to the fraction.

When the sulfate content of the samples was quantified, it was observed that there is no significant difference between F1.1 and F2.0. Although F1.1 and F2.0 both have the same sulfate content, they were precipitated with different volumes of acetone. This is probably because the sulfated polysaccharide conformation interferes during the precipitation process.

**Figure 1:**
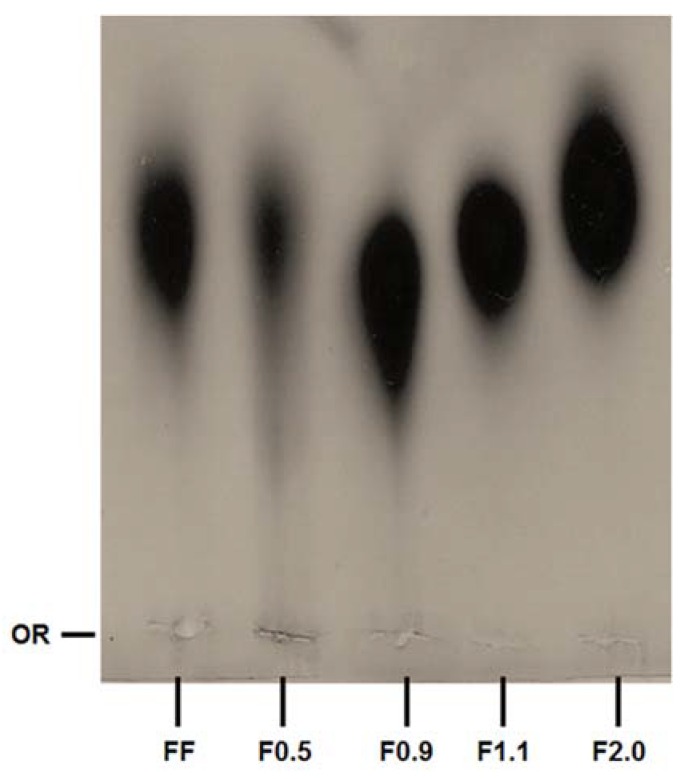
Staining pattern of the polysaccharides after agarose gel electrophoresis, stained with toluidine blue. About 5 µL (50 µg) of each sample was applied in agarose gel prepared in diaminopropane acetate buffer and subjected to electrophoresis, as described in methods. OR—origin. This figure is representative of three separate tests made independently.

To confirm this hypothesis, the fucoidan-rich fractions were subjected to agarose gel electrophoresis in 1,3 diamineacetate (PDA) buffer. Figure 1 shows an agarose gel stained with toluidine blue. It is possible to see that all the fractions have a predominant band. For F0.9, F1.1, and F2.0, the bands display different electrophoretic mobilities. 

### 2.2. Antioxidant Activities

The antioxidant activity of samples was evaluated in vitro by the total antioxidant capacity test (TAC). All samples showed antioxidant activity, though the values were significantly different from each other, particularly for F2.0 fucoidan, which was approximately 400 ± 12.0 equivalents of ascorbic acid. This is nearly double the values obtained for FF and F0.9, which were 189 ± 10.0 and 172 ± 11.0 ascorbic acid equivalents, respectively. The value identified for F1.1 was found to be significantly lower than the two mentioned above (150 ± 8.0 equivalents). The value obtained for F0.5 was around 38 ± 2.0 equivalents, the lowest recorded in this study. There was positive Pearson correlation coefficient between the sulfated content of fractions and TAC (*P* = 0.566).

### 2.3. 3T3-L1 Cell Viability

**Figure 2:**
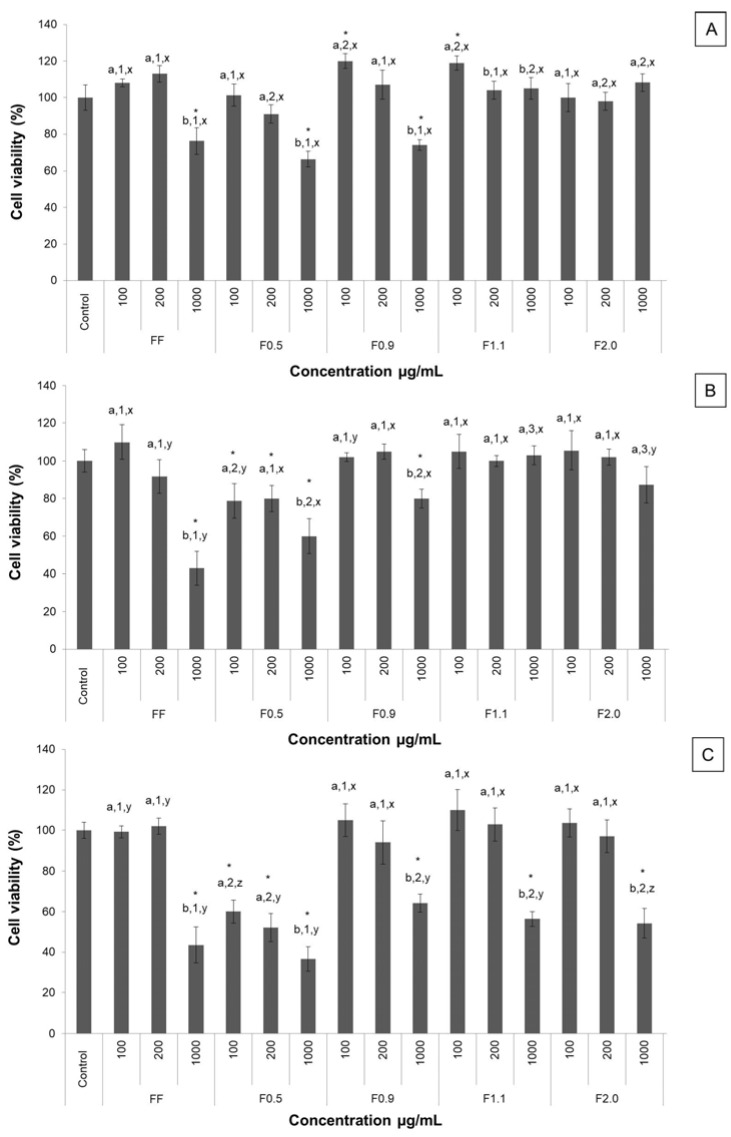
The effects of FF, F0.5, F0.9, F1.1, and F2.0 on 3T3-L1 cell viability. (**A**) 24 h; (**B**) 48 h; (**C**) 72 h. Each value is the mean ± SD of three determinations and from three independent assays. Different letters (^a,b^) indicate a significant difference (*p* < 0.05) between different concentrations of the same sample; Different numbers (^1,2,3^) indicate a significant difference (*p* < 0.05) between the same concentration of each sample; Different letters (^x,y,z^) indicate a significant difference (*p* < 0.05) between the same concentration in different times (24, 48 and 72 h). Asterisks (*) indicate a significant difference (*p* < 0.05) between the concentrations of any sample and the control.

As the 3T3-L1 line (pre-adipocytes) is the main cell model used for the study of adipogenesis, it was first necessary to assess the effects of the samples on the viability of these cells. The results are shown in Figure 2.

Over a period of 24 h (Figure 2A), it was observed that there was a reduction in cell viability (~30%) when the cells were cultured in the presence of FF, F0.5, and F0.9 at the highest concentration tested (1000 µg/mL). A similar effect was observed after 48 h. On the other hand, cytotoxicity (decrease in MTT (3-(4,5-dimethylthiazol-2-yl)2,5-diphenil tetrazolium bromide) reduction by 20%) was also identified using F0.5 at lower concentrations (100 and 200 µg/mL) (Figure 2B). The cytotoxic effects observed with the use of F0.5 was more pronounced after 72 h (Figure 2C), since there was a decrease in the MTT reduction by 40%, 48%, and 64%, using F0.5 in concentrations of 100, 200, and 1000 µg/mL, respectively. The same was also observed with the use of FF, F1.1, and F2.0 (all at 1000 µg/mL), when there was a decrease in MTT reduction of ~55%. Thus, a concentration of 200 µg/mL was selected for use in the following tests relating to the antiadipogenic effects of fucoidans.

### 2.4. Evaluation of the Antiadipogenic Effects of Samples

**Figure 3:**
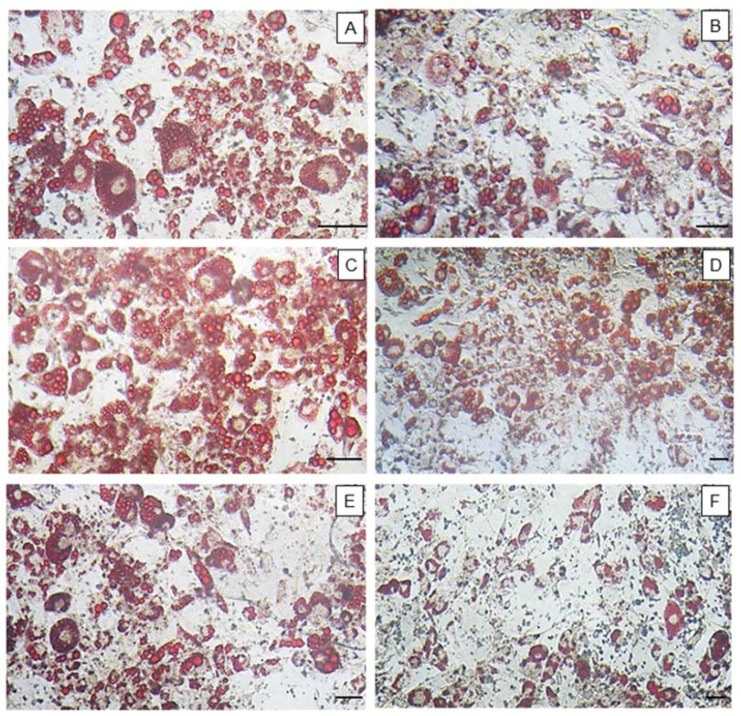
Adipocytes stained with the dye, oil red O. 10× magnification. (**A**) Control; (**B**) FF; (**C**) F0.5; (**D**) F0.9; (**E**) F1.1; (**F**) F2.0. Bar = 60 µm. This figure is representative of three separate tests made independently.

Figure 3 shows that samples had different effects on adipocyte differentiation. As shown in the images below, FF, F1.1, and F2.0 were able to reduce the amounts of neutral lipids within cells, a fact evidenced by the reduced labeling of the cells.

**Figure 4:**
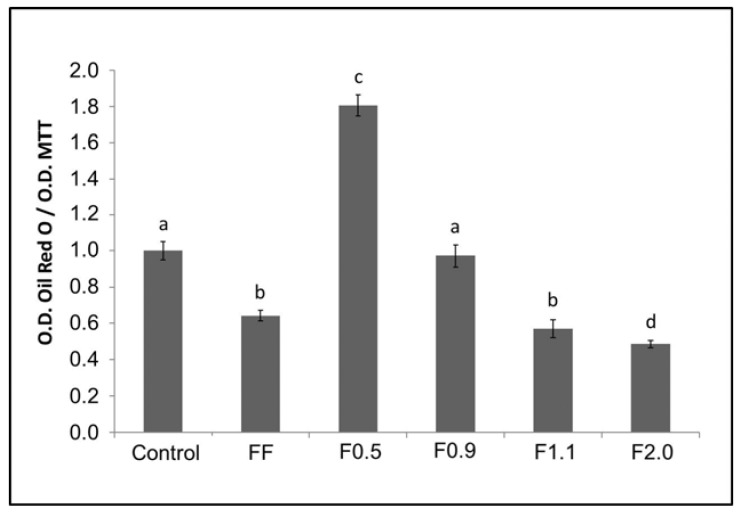
Oil red O content. Each value is the mean ± SD of three determinations and from three independent assays. Different letters (^a,b,c,d^) indicate a significant difference (*p* < 0.05) between the concentration tested (200 µg/mL) of all samples. O.D. (Optical density).

To confirm what was observed by optical microscopy, the oil red O was eluted from the inside of the cells and quantified (Figure 4). As the oil red O dye has an affinity for neutral lipids (triglycerides), the more fat (triglycerides) that is accumulated in the adipocyte the greater the amount of dye within the cell and vice versa. Thus, it was observed that FF induced an approximately 40% reduction of triglyceride in the cells. F2.0 reduced the amount of triglyceride within the adipocytes by approximately 50% and this effect was more pronounced than that observed for FF. An unprecedented result was observed with F0.5, which induced the accumulation of oil red by approximately 80% more than the control group.

**Figure 5:**
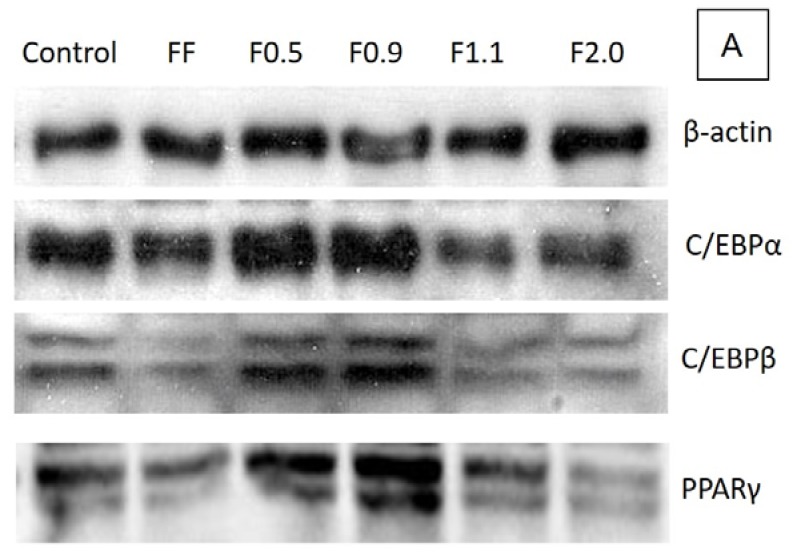

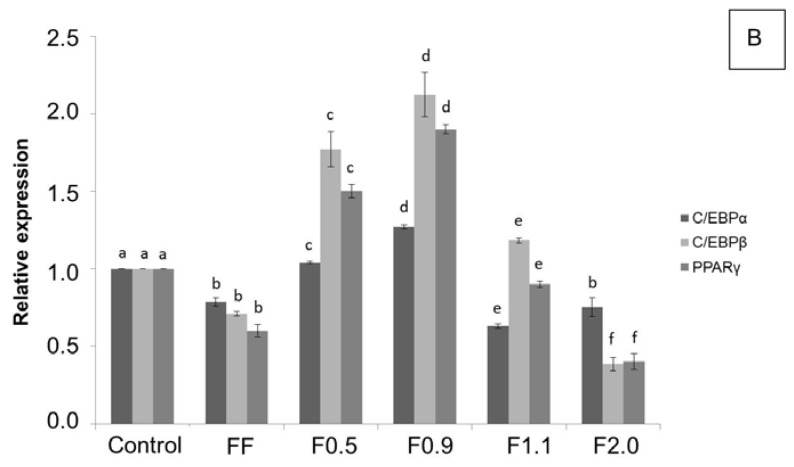
(**A**) The effects of FF, F0.5, F0.9, F1.1, and F2.0 on the expression of adipocyte markers. Equal amounts of protein (50 μg) were used for Western blot analysis, for the detection of β-actin, C/EBPα, C/EBPβ, and PPARγ. These gels are representative of three separate tests made independently; (**B**) Represents the expression relative to the control value. Different letters (^a,b,c,d,e,f^) indicate a significant difference (*p* < 0.05) between the expression of the same marker for different samples. The control corresponds to cells that were not exposed to fucoidans. Each value is the mean ± SD of three determinations and from three independent assays.

To understand the biochemical mechanisms by which these fucoidans act, the expression of key proteins of adipogenesis: C/EBPα, C/EBPβ, and PPARγ of the 3T3-l1 cells was evaluated. Figure 5 shows an immunoblot (Figure 5A) of these proteins. The results obtained from the densitometry of this blot are shown in Figure 5B.

The expressions of these three proteins were altered when the cells were incubated with fucoidans (Figure 5B). F0.5 and F0.9 mainly stimulated the expression of C/EBPβ and PPAR gamma. However, F2.0 reduced the expression of all three proteins evaluated herein, particularly for C/EBPβ and PPARγ, by approximately 55% when compared to the control. In regards to F1.1, we take special note of the particularly strong reduction of C/EBPα expression. These data are in agreement with the oil red test and confirm that F0.5 and F0.9 are adipogenic agents, while F2.0 and F1.1 are antiadipogenic agents.

### 2.5. Evaluation of the Effects of Samples on Lipolysis 

**Figure 6:**
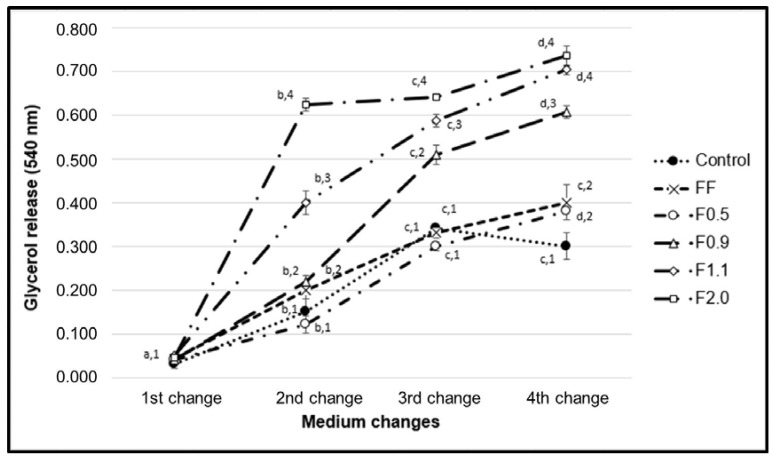
The content of glycerol released into the medium. Each value is the mean ± SD of three determinations and from three independent assays. Different letters (^a,b,c,d^) indicate a significant difference (*p* < 0.05) between the changes of the medium for same sample; Different numbers (^1,2,3,4^) indicate a significant difference (*p* < 0.05) between the same change of the medium in the different samples.

In order to evaluate the potential of the samples to induce lipid hydrolysis in adipocytes, specifically in triglycerides, free glycerol in the culture medium from untreated cells (control), and from cells treated with the fucoidans (200 µg/mL) was quantified. The results are shown in Figure 6.

As illustrated in Figure 6, all samples induced lipolysis. F0.5 was able to induce the mobilization of triglycerides after only 15 days of differentiation, while F0.9 showed a discreet effect by the ninth day of differentiation and after 15 days of culture. Twice as much glycerol in the medium of cells treated with F0.9 than for the control group was found. F1.1 needed only nine days of differentiation to achieve such an effect, i.e., double the lipolysis in comparison with the control group, with this potential being maintained throughout the differentiation process. In turn, F2.0 showed the strongest lipolytic effect, as it was able to increase the breakdown of triglycerides by three times after only nine days of differentiation. Despite the slight decrease in its potential to induce lipolysis during the differentiation process, we also observed 2.5 times more glycerol in the medium of the cells treated with F2.0 than in the control group at the end of the test.

## 3. Discussion

Using differential precipitation with acetone, we acquired four different fractions of commercial fucoidan (FF) from *Fucus vesiculosus*. Other fucoidans were also separated into different fractions by the use of acetone, such as fucoidan from *Spatoglossum schröederi* [18], *Dictyopteris delicatula* [19], and *Dictyota menstrualis* [20]. Acetone separates the different polysaccharides because it competes with them for water molecules. Therefore, the smaller the interaction of the polysaccharide with water, the smaller the amount of acetone that should be added to the solution to precipitate it. Generally, the interactions between sulfated polysaccharides and water partly depend on the amount of charges on the polysaccharide, with the least negatively charged being the first to be precipitated.

Figure 1 shows that fucoidans fractions have different electrophoretic mobilities. In this electrophoresis system, the buffer used (PDA) includes 1,3-diaminopropane in its constitution, which is positively charged at pH 9.0. These positively charged buffers are able to link with the negatively charged groups of the polysaccharides that are exposed, such as sulfates, thus neutralizing them. However, the formation of this complex depends not only on the negatively charged groups of the sulfated polysaccharide, but also on the spatial conformation that the molecule takes in the system, and the effects that it has on how the sulfated polysaccharide exhibits its charged groups. Therefore, the amines do not form complexes with all sulfate groups, but only those that are exposed. In this way, the polysaccharide’s mobility depends on the sulfate groups that have not formed complexes. A classic example is the behavior of chondroitin and heparin, two sulfated polysaccharides that are structurally similar, although heparin is more strongly sulfated. However, in the electrophoresis system with PDA, heparin has a much lower electrophoretic mobility than chondroitin [21]. In a previous study it was shown that commercial fucoidan was composed of fucose, glucose, galactose, mannose, xylose, glucuronic acid and sulfate [17]. Of these monosaccharides we find neither glucose nor mannose in FF. This is probably because the composition of commercial fucoidans should vary. So, we also did not find these two monosaccharides in the fractions. In addition, the relative proportion of glucuronic acid, xylose and galactose was different in each sample. Overall, these data lead us to propose that we have obtained four different fucoidan-rich fractions from *F. vesiculosus*.

Qi et al. [22] suggested that the antioxidant activity of fucoidan is related to the degree of sulfation: the more sulfated the polysaccharide, the more active it is. However, the data from the TAC test did not agree with those suggested by these authors. In addition, there was a weak Pearson correlation coefficient between the sulfated content of fractions and TAC. Furthermore, the data submitted by various authors that evaluated the antioxidant capacity of fucoidans obtained from other brown seaweed [19,23,24,25] indicate that the structure of fucoidans, as well as the exposure of the oxidizable groups along the molecule, are more important factors for the antioxidant capacity of these polymers than the number of sulfate groups.

The antiadipogenic activity of the fucoidan (FF) from *F. vesiculosus* had previously been reported [9]. However, the fucoidan fraction constituents of FF have not yet been evaluated in isolation for their antiadipogenic potential. In this work, we observed that these fractions had different effects on adipocyte differentiation.

Since oil red O has an affinity for neutral lipids (triglycerides), the greater the amount of fat (triglycerides) accumulated within adipocytes, the more dye will be observed within that cell, and vice versa. FF promoted a reduction of triglycerides in cells of around 40%. This value was highly similar to that observed by other authors, for example by Kim et al. [26]. In 2009, they reported that a commercial fucoidan was able to reduce the incorporation of oil red O by adipocytes in 39.7%, at a concentration of 200 μg/mL [9]. An unprecedented result was observed for F0.5 and F0.9, which induced the accumulation of triglycerides to approximately 80% more, compared to the control group. On the other hand, F2.0 fucoidan reduced the amount of triglycerides within adipocytes by approximately 50%, with this effect being more pronounced than that observed using FF.

To better understand the biochemical mechanism by which these fucoidans act, we verified the expression of key regulatory proteins of adipogenesis: C/EBPα, C/EBPβ, and PPARγ, as it has been reported that commercial fucoidan affects these enzymes [9,26]. In agreement with the results of the oil red O test, F0.5 and F0.9 caused an acute increase, mainly in the expression of C/EBPβ and PPARγ, in comparison to the control, confirming that these two compounds are adipogenic agents. However, F1.1 and F2.0 caused a reduction in the expression of these proteins, which in turn led to a reduction in the amount of triglycerides inside the adipocytes, as observed in the oil red O test.

The effects caused by fucoidans on gene expression and adipogenesis regulatory proteins have been previously reported in the literature, corroborating our results. FF was reported to reduce preadipocyte differentiation in adipocytes by reducing the expression of certain genes, including *PPARγ* [26], and *C/EBPα*, by 22.6% and 17.6%, respectively [9].

F1.1 and F2.0 were able to induce the release of triglycerides from the interior of the adipocytes (Figure 6). The molecular pathways by which these fucoidans act was not suggested, but fucoidan is known to stimulate the activity of hormone-sensitive lipase (LSH) [8]. LSH regulates lipolytic activity within adipocytes, and the phosphorylation of this enzyme leads to its activation with consequent hydrolysis of the triglycerides stored inside these cells, which subsequently release their glycerol and fatty acids [27]. Therefore, we believe that the LSH activation pathway may be the target of fucoidan activity demonstrated in our work. We intend to investigate this hypothesis in future work.

Anti-obesity drugs, besides causing a series of undesired effects, may possibly contribute to the onset of cardiovascular diseases [28]. Thus, the search for new agents that are effective against obesity and present lower health risks remains a priority [29]. We believe that *Fucus vesiculosus* fucoidan and its fractions could potentially be used to develop future treatments for obesity. We hope that our work will contribute to this end.

## 4. Materials and Methods

### 4.1. Materials

Commercial fucoidan extracted from *Fucus vesiculosus*, monoclonal mouse anti-β-actin antibody, 3-Isobutyl-1-methylxanthine, dexamethasone, insulin, and free glycerol reagent were obtained from Sigma-Aldrich^®^ (St. Louis, MO, USA). Sodium bicarbonate, culture media components [minimum essential Dulbecco’s modified Eagle medium (DMEM)], non-essential amino acids, fetal bovine serum, sodium pyruvate, and phosphate buffered saline [PBS] were purchased from Invitrogen Corporation (Burlington, ON, USA). Monoclonal rabbit anti-CEBPα, anti-CEBPβ, anti-PPAR-γ and anti-rabbit and anti-mouse horseradish peroxidase-conjugated secondary antibodies were obtained from Cell Signaling Technology (Beverly, MA, USA). Other solvents and chemicals used in this study were of analytical grade.

### 4.2. Cell Culture

3T3-L1 preadipocyte cells were purchased from the cell bank of Rio de Janeiro, RJ, Brazil (CR089-BCRJ/UFRJ), and maintained with 10% fetal bovine serum (FBS)/DMEM containing 4.5 g/L glucose, 100 U/mL penicillin, 0.1 mg/mL streptomycin, and 0.25 mg/mL amphotericin B at 37 °C in 5% CO_2_ incubator.

### 4.3. Obtaining Different Populations of FF from F. vesiculosus

Four grams of FF were solubilized into 0.25 M sodium chloride. To this solution was added increasing volumes of acetone to precipitate the different fucoidans. To obtain fucoidan F0.5, for example, 0.5 volumes of ice-cold acetone was added to this solution slowly and under gentle agitation, and then held at 4 °C for 12 h. The precipitate formed was collected by centrifugation at 8000× *g* for 15 min at 4 °C. Acetone was added to the supernatant until a precipitated material appeared. Like this, we used the acetone volumes of 0.9, 1.1 and 2.0, calculated from the initial solution, and the operations repeated as above.

### 4.4. Determination of Sulfate Content and Monosaccharide Composition of Fucoidans

In order to determine the amount of sulfate, the fucoidan samples were hydrolyzed (4 N HCl for 6 h at 100 °C), and then the sulfate amount was given by turbidimetry at 500 nm by the gelatin/barium method as described early [23]. Sodium sulfate was used as standard.

To determine the best hydrolysis condition, the polysaccharides were hydrolyzed with 0.5, 1, 2, and 4 M, respectively, for various lengths of time (0.5; 1; 2; and 4 h), at 100 °C and the amount of reducing sugars of each condition was determined as described early [23]. Hydrolysis with 2 M HCl for 2 h producing the best reducing sugar yields.

After acid hydrolysis (2 M HCl for 2 h, 100 °C), sugar composition was determined by a LaChrom Elite^®^ HPLC system from VWR-Hitachi (Hitachi Co., Tokyo, Japan) with a refractive index detector (RI detector model L-2490, Hitachi Co., Tokyo, Japan). A LichroCART^®^ 250-4 column (250 mm × 40 mm, Merck, Darmstadt, Germany) packed with Lichrospher^®^ 100 NH_2_ (5 µm, Merck, Darmstadt, Germany) was coupled to the system. The sample mass used was 0.2 mg and analysis time was 25 min. The following sugars were analyzed as references: arabinose, fructose, fucose, galactose, glucose, glucosamine, glucuronic acid, mannose, and xylose.

### 4.5. Agarose Gel Electrophoresis

Agarose gel electrophoresis of the fucoidans was performed in 0.6% agarose gel (7.5 cm × 10 cm × 0.2 cm thick) prepared in 0.05 M 1.3-diaminopropane acetate buffer pH 9.0, as previously described [20]. Aliquots of the samples (50 μg) were applied to the gel and subjected to electrophoresis. The gel was fixed with 0.1% cetyltrimethylammonium bromide solution for 2 h, dried, and stained for 15 min with 0.1% toluidine blue in 1% acetic acid in 50% ethanol. The gel was then destained with the same solution without the dye.

### 4.6. Determination of Total Antioxidant Capacity (TAC)

This assay is based on the reduction of Mo^6+^ to Mo^5+^ by samples and subsequent formation of a green phosphate-molybdate complex at acid pH [19,23]. Tubes containing extracts and reagent solution (0.6 M sulfuric acid, 28 mM sodium phosphate and 4 mM ammonium molybdate) were incubated at 95 °C for 90 min. After the mixture had cooled to room temperature, the absorbance of each solution was measured at 695 nm against a blank. The TAC was accounted in ascorbic acid milligrams/sample grams, described as equivalent of ascorbic acid.

### 4.7. MTT Assay

The 3T3-L1 cell capacity to reduce MTT after fucoidan exposure was evaluated in vitro according to the method described earlier [19]. This method is based on the reduction of MTT to formazan crystals by living cells. Briefly, 5 × 10^3^ 3T3-L1 cells/well were plated in a 96-well plate. For which well 100 μL DMEM medium with 10% FBS was the final volume used. The cells grew up (95% air, 5% CO_2_, 37 °C) until 80% confluence and then the medium was replaced by a DMEM without FBS, followed by incubation for another 24 h in order to stimulate cells to enter in G0 phase. After, the medium was replaced by a DMEM with 10% FBS in the presence of different fucoidans (from 100 to 1000 μg/mL) or absence (control) for 24, 48 or 72 h. At the end of the incubation period, the medium was replaced by a new DMEM without FBS added to 5.0 mg/mL of MTT, followed by incubation for 4 h at 37 °C. The medium was removed and formazan crystals were dissolved with 100 µL of 95% ethanol. After 15 min shaking in a rocking shaker, absorbance was read (570 nm) in a microplate spectrophotometer (Biotek, Winooski, VT, USA). As a negative control, cells were cultivated only with DMEM with 10% FBS. Results were expressed in the percentage of MTT reduction, as in Equation (1).

Percentage MTT Reduction = (Absorbance of sample/Absorbance of control) × 100(1)

### 4.8. Adipocyte Differentiation 

For adipocyte differentiation the cells were cultured in 24-well plates or 10 mm culture plate containing DMEM medium enriched with 10% FBS and, after reaching 80% confluence, these cells were induced to differentiate by the addition of the adipocyte differentiation cocktail: DMEM, 10% FBS, 1 μM dexamethasone, 0.5 mM 3-isobutyl-1-methylxanthine (IBMX) and 10 μg/mL insulin. After 72 h, the induction medium was replaced by the adipocyte maintenance medium, which consisted of DMEM, 10% FBS and 10 μg/mL insulin. The maintenance medium was changed every 3 days until completing 15 days of differentiation.

### 4.9. Oil Red O Staining

For oil red O staining, cells were initially induced to differentiate into adipocytes, as previously mentioned, in the absence (control) or presence of fucoidans (200 μg/mL). For this, these samples were added to the differentiation medium. The cells were exposed to fucoidan only in the first three days. After 15 days of differentiation, the cells were washed twice in PBS, fixed with a solution of formaldehyde (3.7%) in PBS for 1 h, washed three times in water, dried and stained with oil red O for 1 h. Excess dye was removed by washing with water. Images of the cells were captured using an optical microscope (TE-Eclipse 300, Nikon, Melville, NY, USA). We performed three different experiments. Subsequently, the dye was eluted from the cells with 100% isopropanol and quantified by measuring the absorbance at 520 nm. The results of the differentiated cells in the presence of the samples were compared with those obtained for the control cells (100% adipocitary differentiation).

### 4.10. Analysis of the Expression of Adipogenic Markers by Western Blotting

3T3-L1 cells were plated in 100 mm plates and stimulated to differentiate into adipocytes (as described above) in the presence or absence of the 200 μg/mL samples (FF; F0.5; F0.9; F1.1; F2.0). After 15 days of differentiation the cells were suspended in the lysis buffer [50 mM Tris-HCl (pH 7.4); 1% Tween 20; 0.25% sodium deoxycholate; 150 mM NaCl; 1 mM EGTA; 1 mM Na_3_VO_4_; 1 mM NaF; and protease inhibitors] to obtain the protein extract. The protein content was determined using the Bradford method (1976). The control corresponds to cells that were not exposed to fucoidans. For each sample, 30 μg of protein were subjected to sodium dodecyl sulfate polyacrylamide gel electrophoresis (SDS-PAGE) with subsequent transfer to polyvinylidene difluoride (PVDF) membrane (Millipore, Bedford, MA, USA). The membranes were blocked with skim milk (1%) or albumin (1%) and incubated for 18 h at 4 °C with appropriate primary antibody (β-actin, C/EBPα, C/EBPβ and PPARγ), in a dilution of 1:1000. After incubation with appropriate peroxidase-conjugated (anti-mouse or anti-rabbit) secondary antibody (1:1000 dilution), the detection was performed by chemiluminescence. β-actin was used as an internal control to evaluate the uniformity of protein loading and transfer. The bands were visualized with a ChemiDoc imaging system (Bio-Rad, Hercules, CA, USA) and quantified by ImageJ software ver.1.51k (National Institutes of Health, Bethesda, MD, EUA).

### 4.11. Statistical Analysis

All data from the experiments were expressed as mean ± standard deviation (*n* = 3) and from three independent assays. The pictures (Figure 1, Figure 3 and Figure 5A) are representative of three separate tests made independently. Statistical analysis between FF and fucoidan´s fraction was done by analysis of variance (ANOVA). The Student-Newman-Keuls post-test (significance was set at *p* < 0.05) was applied to prove some similarities found by ANOVA. All experiments were done in triplicate and data refer to the average of three experiments done independently.

The Pearson correlation coefficient was calculated for the sulfate and the data from the CAT test obtained with fucoidan fractions. 

Statistical analysis and Pearson correlation coefficient were performed using GraphPadPrism^®^ software version 5.0, 2014 (GraphPad, La Jolla, CA, USA).

## 5. Conclusions

In this paper, four different fucoidan-rich fractions (F0.5/F0.9/F1.1/F2.0) of commercial fucoidan from *F. vesiculosus* were obtained using differential precipitation with acetone. Chemical analyzes showed fucose was the major component present in all fractions whereas the relative amounts of other monosaccharides vary according to the fraction. Monosaccharide composition, agarose gel electrophoresis, and antioxidant data showed that each fraction had a different type of fucoidan. F0.5 and F0.9 have an adipogenic effect because they stimulated the lipid accumulation in 3T3-L1 adipocytes through up-regulation of C/EBPα, C/EBPβ, and PPAR gamma. On the other hand, F1.0, and F2.0 have antiadipogenic activity. In addition, all samples had lipolytic action, especially F2.0, which tripled the amount of glycerol in the cellular medium. Among these, F2.0 showed a marked effect on the attenuation of lipid accumulation, antioxidant, and lipolytic activities, and we thus recommend it as a natural antiadipogenic agent for several biotechnological applications.

## Figures and Tables

**Table 1:** Chemical composition of fucoidan (FF) and its fractions. Fuc: fucose; Gluc acid: glucuronic acid; Gal: galactose; Xyl: xylose; Man: mannose; Gluc: glucose; n.d—not detected. Different letters (^a,b,c,d^) indicate a significant difference (*p* < 0.05) between the samples. Each value is the mean ± standard deviation (SD) of three determinations and from three independent assays.

Sulfated Polysaccharides	Yield (%)	Sulfate (%)	Molar Ratio					
			Fuc	Gluc Acid	Gal	Xyl	Man	Gluc
FF	-	23.70% ± 0.04 ^a^	1.0	0.7	0.4	0.2	n.d	n.d
F0.5	4.5	12.70% ± 0.08 ^b^	1.0	0.2	0.3	0.5	n.d	n.d
F0.9	35.2	17.40% ± 0.02 ^c^	1.0	0.8	0.2	0.6	n.d	n.d
F1.1	22.0	20.30% ± 0.05 ^d^	1.0	0.2	0.4	0.2	n.d	n.d
F2.0	38.3	20.40% ± 0.04 ^d^	1.0	0.1	0.3	0.1	n.d	n.d
